# Effectiveness of a 5-Week Virtual Reality Telerehabilitation Program for Children With Duchenne and Becker Muscular Dystrophy: Prospective Quasi-Experimental Study

**DOI:** 10.2196/48022

**Published:** 2023-11-15

**Authors:** María Rosa Baeza-Barragán, Maria Teresa Labajos Manzanares, Mercedes Cristina Amaya-Álvarez, Fabián Morales Vega, Judit Rodriguez Ruiz, Rocío Martín-Valero

**Affiliations:** 1 CTS-1071 Research Group, Department of Physiotherapy, Faculty of Health Science, University of Málaga, Málaga, Spain; 2 Physiotherapy Unit, Gabinete Reto, Málaga, Spain; 3 Department of Physiotherapy, Fernando Pessoa University, Canary Islands, Spain; 4 Department of Physiotherapy, Poniente University Hospital, Almería, Spain

**Keywords:** Duchenne muscular dystrophy, telerehabilitation, virtual reality, physical therapy, muscular dystrophy, mutation, muscle, degeneration, telehealth, motor function, digital health intervention, Becker muscular dystrophy, VR

## Abstract

**Background:**

Duchenne muscular dystrophy (DMD) and Becker muscular dystrophy (BMD) are neuromuscular diseases. DMD is the most prevalent in children. It affects dystrophin production, reducing the patient’s mobility and quality of life. New technologies have become a part of physical therapy in DMD and BMD. During the COVID-19 pandemic, conducting telerehabilitation through virtual reality–based games could help these children maintain their physical abilities.

**Objective:**

This study examined if the use of a virtual platform in a multimodal intervention program changes the results of the 6-minute walk test (6MWT) in children with DMD and BMD. The main objective was to test whether children with DMD and BMD obtain different results on the 6MWT after completing 10 telerehabilitation treatment sessions. The secondary objective was to measure whether other specific motor scales also produce different results after the 10 defined sessions.

**Methods:**

This was a descriptive, open, and quasi-experimental study with a prospective A-B (control-intervention) design. A sample of 12 participants who fulfilled the inclusion criteria followed the program for 5 weeks with 10 telerehabilitation sessions. During the sessions, the participants used virtual reality glasses to train for the treatment goals. All participants were assessed in person before and after the intervention. Analysis was performed using R software according to the different functional assessments performed for each test.

**Results:**

The participants showed a 19.55-meter increase in the 6MWT. Motor function also remained stable according to other scales used to assess it. The North Start Ambulatory Assessment scores were stable in both treatment conditions (*P*=.20). Furthermore, the timed up and go test results were 0.1 seconds faster in the telerehabilitation condition, and the Motor Function Measure in all of the 3 dimensions showed no significant differences (*P*=.08). Finally, the Effort Perception Infant scale showed that during the training, fatigue increased in the middle and decreased by the end of the sessions, but the perception throughout the sessions was lower even as the exercise intensity increased.

**Conclusions:**

There were no differences between conventional and telerehabilitation treatments, so the telerehabilitation tool could be used without harming children with DMD and BMD, facilitating their access to therapies and stimulating learning to maintain their functional capacity. Therefore, telerehabilitation in general may be helpful in maintaining motor function in children with DMD and BMD. The learning effect helped reduce the feeling of fatigue in the children during the program.

## Introduction

### Background

Duchenne muscular dystrophy (DMD) and Becker muscular dystrophy (BMD) are rare neuromuscular diseases caused by a mutation in the gene that produces dystrophin, which is responsible for maintaining muscle properties [1]. The lack of dystrophin leads to the progressive degeneration of muscle fibers, which become connective tissue and fat [2]. The worldwide prevalence is approximately 0.5 per 10,000 male individuals for DMD and 1 per 3500 male individuals for BMD [3]. The loss of ambulation occurs at a median age of 12 years, and ventilation starts at about the age of 20 years, making it a disabling disease with severe motor sequelae and respiratory and cardiac problems, with a high cost to health [4].

Maintaining function as long as possible is the primary goal of physical therapy for these conditions. To start intervention from the beginning of the diagnosis or to document the evidence of a motor delay, physical therapy is carried out using techniques such as contracture and posture management, as well as aerobic, proprioceptive, coordination, balance, and respiratory work [5]. Children with DMD and BMD have associated disorders such as autism, speech limitations, and behavioral problems in most cases, so the proposed treatment was aimed at treating these associated disorders [3].

The global situation due to COVID-19 has led health professionals to carry out interventions through video calls to support families. The impact of the pandemic, confinement duration, and health care changes could affect the mental health of young people with intellectual disabilities and their environment. Their behavior could be aggravated, and mental disorders could appear [6]. Furthermore, the characteristics of children at advanced stages of DMD and BMD, such as muscle weakness, impaired cough reflex, mucus in the airways, cardiac deterioration, and the very early intake of corticosteroids, may predispose them to severe COVID-19 [5]. A major difference between DMD and BMD is that BMD progresses slower and has a later onset than DMD [5].

The World Confederation for Physical Therapy, in its report on the regulation of clinical practice through digital platforms, summarizes the following benefits: this practice facilitates connection, reduces barriers and waiting times, increases safety, reduces mobility problems, improves user independence, improves flexibility, and lowers costs of care [7]. During this time, the effectiveness of active video games, a game system where the player’s movements through the game space are displayed on a television screen, and virtual reality (VR) games, which provide an artificial 3D environment using VR technology, has been demonstrated in the physical rehabilitation of children with disabilities at home [8].

As part of telehealth, telerehabilitation uses telecommunications technologies to control or monitor remote rehabilitation. Its objective is to increase accessibility and continuity of care for vulnerable, geographically remote populations with disabilities, thus saving time and resources for health care providers [9]. In addition, telerehabilitation implementation is feasible, it can be offered to diverse groups of patients, and therapists accept it as an effective model of care for children [10].

The use of VR in physical therapy programs for children can provide a personalized and flexible treatment by allowing us to integrate the child’s preferences into the intervention program, improve attention and motivation, and increase sensory feedback [11]. Despite the effectiveness of the programs in general, there are few studies about children with DMD and BMD.

Previous studies have used accelerometers to measure functional tasks and the effectiveness of dynamic arm support at home in young patients with DMD [12,13]. Web-based platforms have also been used to demonstrate respiratory exercises to relatives and provide a satisfaction survey to measure the participation and acceptance of the program [14].

### Objectives

The main objective of this study was to test whether children with DMD and BM obtain different results on the 6-minute walk test (6MWT) [15] after completing a multimodal telerehabilitation program of 10 sessions over 5 weeks with VR glasses as a part of the treatment. As a secondary objective, other specific motor scales were used to measure changes after receiving the 10 defined sessions. These scales were the North Star Ambulatory Assessment (NSAA) [16], timed up and go test (TUG) [17], Motor Function Measure (MFM) [18], and Effort Perception Infant (EPInfant) [19], which will be described in the *Methods* section.

The purpose of this study was to examine if the use of a virtual platform in a multimodal intervention program changes the results of the 6MWT in children with DMD and BMD.

## Methods

### Study Design

This was a descriptive, open, and quasi-experimental study using an A-B design with a prospective control condition and an intervention condition. It was carried out nationally, according to a multicentric scheme.

The principal investigator conducted the sessions with all participants. In all, 3 researchers from the Canary Islands, Madrid, and Almería conducted the pre- and poststudy assessments of the variables. The interviews were conducted by the associate researcher in each department.

All caregivers were notified by email and WhatsApp 1 day before the session was held, to ensure the continuity of the study and its frequency of 2 weekly sessions for 5 weeks.

A control condition and an intervention condition were defined for the entire sample. Both results were compared, and each child was only compared to their own results. Upon the completion of the multimodal physical therapy program, the main researcher performed an outcome assessment and functional assessment of the intervention face to face.

The following sections describes the training carried out with the study participants. Over 5 weeks, the participants passed a pretraining assessment (control condition) and then attended ten 30-minute physiotherapy sessions (intervention condition).

### Control Condition

The participants first underwent the control condition, where they performed the functional tests by following a conventional physical therapy program without the use of VR glasses.

### Intervention Condition

The participants then underwent the intervention condition of ten 30-minute sessions, which included self-stretching, yoga, aerobic work, breathing and relaxation, mindfulness, and training with VR glasses (Table 1). We used sources on the internet such as videos on YouTube [20] to carry out the guided exercises, as well as music on Spotify. Visual and musical support during the sessions was one of the key factors for the child to want to participate and maintain their attention during the treatment. Cardboard VR glasses sent to each of the participants were also used as support.

**Table 1.** Exercises performed during the telerehabilitation sessions.

Type of exercise	Material resources	Time	Example
Aerobic	Video game Dancing Walking Therapist	Gradual increase in exercise duration from 3 min to 6 min	While watching a video of a walk, the child keeps walking in place.
Respiratory	Video story Therapist	10 min	The reading of a visual story with music is used as an opportunity for breathing exercises, prolonged expirations, strengthening of the muscles involved in breathing, deep diaphragmatic inspirations, etc.
Self-stretching	Music Therapist Yoga Videos	4-5 min	A guided self-stretching session is performed with the therapist following the rhythm of the music.
Relaxation	Mindfulness Videos	3 min	Using mindfulness as a tool, we integrate a child’s ability to stop, relax, breathe, and disconnect through listening.
VR^a^	VR glasses VR videos	6-8 min	Through the VR glasses, the child is required to perform various activities such as swimming, crouching, walking on the spot, or breathing by viewing a VR video.

a VR: virtual reality.

The participants used the Zoom (Zoom Video Communications) video call platform to carry out a program of web-based gamification activities. The multimodal physiotherapy treatment, adapted to a virtual platform, was aimed at global work of the child at different levels (respiratory, aerobic, and mental levels), postural re-education, and self-stretching; in addition, gamification, dance, yoga, and meditation were used as tools within the physiotherapy framework [21]. The child performed the intervention alone or with their caregivers. A physiotherapist guided all sessions. Neither the participants nor the caregivers received training or have knowledge of how they were going to perform the exercises.

### Recruitment

The recruitment of participants started in March 2021 and was completed in October 2022. Recruitment took place at each child’s referring physiotherapy center. To be included in the study, the principal investigator was contacted directly. This investigator’s contact information was provided in the study information sheet. Participants who met the inclusion requirements of the study were contacted by a phone call as the first contact and for the clarification of doubts.

The technique used to select the participants was of an intentional, nonprobabilistic nature. The selection was made according to the inclusion and exclusion criteria, and all who met them were included.

Due to the low recruitment capacity (scarce eligible population), convenience sampling was carried out. The allocation process was not randomized. A random sample of 12 individuals was considered significant enough to estimate, with a 95% confidence and an accuracy of within 80 meters, the population mean values with an expected SD of around 134.35 meters. The SD of the control treatment and telerehabilitation was 95 meters. The predicted withdrawal rate was 5% or below. The estimate was made using the GRANMO calculator (Institut Municipal d’Investigació Mèdica) [22].

### Inclusion and Exclusion Criteria

Participants were children between the ages of 5 and 15 years with muscular dystrophy (DMD or BMD) who could walk and follow the web-based intervention program; were diagnosed according to the International Classification of Diseases, 10th Revision (code G71-01); and had an NSAA score greater than 20 [23-25]. The characteristics of the participants are shown in Multimedia Appendix 1.

Participants who did not walk were excluded, as well as those with associated heart disease, any fracture or sprain, heart rate >120 beats per minute, or oxygen saturation <89% [15,26,27] and those who did not have internet literacy.

### Primary and Secondary Outcome Measures

The primary outcome measure was the change in the 6MWT (time frame: from baseline up to 5 wk) [15]. The 6MWT is a well-established outcome measure in various diseases. It is accurate, reproducible, easy to administer, and well tolerated. The 6MWT is a robust assessment tool for clinical trials, given its ability to assess ambulation quantitatively in a controlled setting.

The following secondary outcome measures were also considered:

- The EPInfant is a perceived child effort measurement scale (time frame: to study completion, 5 wk on average) [19]. It shows 11 numerical descriptors (from 0 to 10), a total of 5 verbal descriptors placed at every 2 intensity levels, and a set of illustrations depicting a boy running at increasing intensities along a bar scale of increasing height following an exponential-type slope from left to right. Higher values correspond to worse results.
- The NSAA is functional scale for children with DMD (time frame: from baseline up to 5 wk) [16]. It is expressed in points and shows the acquisition or loss of functions. Although children with DMD can generally show recognizable adaptations to activities due to the underlying progressive muscle weakness, they may modify their activities to achieve functional goals in slightly different ways. In general, activities receive the following scores: 2 (“Normal”)=no obvious modifications to the activity; 1=modified method that achieves the goal independently of physical assistance from others; and 0=unable to achieve the goal independently. The best score is 34 points.
- The MFM measures motor performance (time frame: from baseline to 5 wk) [18]. It has 3 dimensions: D1, standing and transfers (13 items); D2, axial and proximal motor function (12 items); and D3, distal motor function (7 items; 6 of which evaluate the upper extremities).
- The TUG is expressed in seconds (time frame: from baseline to 5 wk) [17]. It shows the time it takes children to stand up, walk 3 meters, return to the seat, and sit down.
- The Vignos Scale ranges from 1 to 10 and assesses lower-extremity function. A score of 1 refers to being able to walk without assistance and 10 refers to being confined to a bed. It is embedded within the MFM data collection [18].

The CONSORT (Consolidated Standards of Reporting Trials) flow diagram of participants’ recruitment and progress through the phases of the trial is shown in Multimedia Appendix 2.

### Data Analysis

The analysis was organized according to the different functional assessments performed for each test. Analysis was performed using R (R Foundation for Statistical Computing) software. Scores were calculated using the corresponding guidelines. Each variable was analyzed to measure the motor function used: 6MWT, NSAA, TUG, and MFM. In addition, we analyzed the perceived fatigue with EPInfant, including the variation during the course of the sessions and the relationship with the rest of the variables.

The correlation between fatigue and motor function was assessed using the Pearson correlation coefficient. Paired tests were used to account for the inherent dependence and correlation among the variables. The 2-tailed paired *t* test was used for variables conforming to a normal distribution, whereas the Wilcoxon signed-rank test was used for variables that did not adhere to normal distribution assumptions. The Anderson-Darling and Fligner-Killeen tests were used to evaluate normality and homoscedasticity assumptions, respectively. The Friedman test was used when the evaluation spanned beyond 2 distinct time points. In cases where statistically significant relationships were detected (*P*<.05), we applied the Bonferroni correction method to rigorously examine post hoc relationships. Additionally, 2 linear mixed-effects models (random intercepts) were fitted to evaluate child stress. The first model, M1, does not incorporate an interaction between time and sessions and can be represented as:


scoreij​∼N(μ,σ2)=αj​[i]+β1​(timeMiddle)+β2​(timeEnd)+β3​(Sessions)∼N(μαj​​,σαj​2​)

The second model, M2, incorporates an interaction and is expressed as:


scoreij​∼N(μ,σ2)=αj​[i]+β1​(timeMiddle)+β2​(timeEnd)+β3​(Sessions)+β4​(Sessions×timeEnd)+βs5​(Sessions×timeEnd)∼N(μαj​​,σαj​2​)

The above equations apply for patient *j*=1...*j*.

### Ethical Considerations

The project’s development followed the World Medical Association Declaration of Helsinki in 1964 and the ratifications of the subsequent assemblies on ethical principles of medical research involving human participants. The researchers in this study agreed that all clinical data collected from study participants would be separated from personally identifiable data to ensure patient anonymity. The researchers collected the patients’ clinical data in the study-specific data collection notebook. Each data collection notebook was coded and pseudonymized, thus protecting the patient’s identity.

It was guaranteed that the treatment, communication, and transfer of personal data of all participants complied with the provisions of Organic Law 3/2018, of December 5, on protecting personal data and guaranteeing digital rights. The participants were informed about their rights to access, cancel, or oppose the use of their data by emailing the researchers. There was no compensation for participation.

This trial has the approval of the Andalucía Ethics Committee with Portal de Ética de la Investigación Biomédica de Andalucía code 0107-N-20.

All participants were informed, and all signed a paper-based informed consent form in person.

## Results

### Primary and Secondary Outcome Measures

#### Overview

Table 2 describes the results for each variable.

**Table 2.** Summarized means and SDs of the variables (N=12).

Variable	Conventional treatment, mean (SD)	Telerehabilitation, mean (SD)	Difference, mean (SD)
6MWT^a^	408.24 (115.61)	427.79 (134.40)	19.55 (43.83)
NSAA^b^	0.76 (0.18)	0.71 (0.21)	–0.05 (0.11)
Vignos Scale	2.08 (0.79)	2.25 (1.06)	0.17 (0.39)
TUG^c^	7.70 (3.49)	7.60 (3.75)	–0.10 (0.90)
Total MFM^d^	0.89 (0.10)	0.85 (0.14)	–0.04 (0.13)

a 6MWT: 6-minute walk test.

b NSAA: North Star Ambulatory Assessment.

c TUG: timed up and go test.

d MFM: Motor Function Measure.

#### 6MWT Outcomes

After completing the conventional treatment and then changing to telerehabilitation, the patients walked an average of 19.55 (95% CI –8.30 to 47.40) meters more. This increase was not significant against the distance walked after the conventional treatment (*P*=.15; Table 3).

**Table 3.** Results of the 6-minute walk test (6MWT)^a^ (N=12).

6MWT (m)	Conventional treatment	Telerehabilitation	Difference
Value, mean (SD)	408.24 (115.61)	427.79 (134.40)	19.55 (43.83)
Value, median (IQR)	364.50 (323.62 to 459.38)	375.50 (346.88 to 428.75)	15.00 (–5.25 to 37.50)
Value, range	294.00 to 630.00	324.00 to 750.00	–51.50 to 120.00
Value, 95% CI	334.79 to 481.70	342.40 to 513.19	–8.30 to 47.40

a Paired *t* test: *t*_11_=–1.545, *P*=.15 (not significant).

#### NSAA Outcomes

The NSAA scores were kept stable at around 73%. Although the score for telerehabilitation decreased (5%, 95% CI –13% to 2%), this decrease was not statistically significant with respect to the conventional approach (*P*=.20; Table 4).

**Table 4.** Results of the North Star Ambulatory Assessment (NSAA)^a^ (N=12).

NSAA score	Conventional treatment	Telerehabilitation	Difference
Value, mean (SD)	0.76 (0.18)	0.71 (0.21)	–0.05 (0.11)
Value, median (IQR)	0.85 (0.63 to 0.88)	0.68 (0.62 to 0.89)	–0.03 (–0.09 to 0.01)
Value, range	0.44 to 1.00	0.32 to 0.97	–0.26 to 0.09
Value, 95% CI	0.65 to 0.87	0.57 to 0.84	–0.13 to 0.02

a Paired Wilcoxon test: *V*=40.000; *P*=.20 (not significant).

#### TUG Outcomes

The time used in the TUG during the telerehabilitation period was 0.1 (95% CI –0.67 to 0.49) seconds shorter than that during the conventional period. This decrease was not statistically significant (*P*=.72; Table 5).

**Table 5.** Results of the timed up and go test (TUG) (N=12).

TUG (s)	Conventional treatment	Telerehabilitation	Difference
Value, mean (SD)	7.70 (3.49)	7.60 (3.75)	–0.10 (0.90)
Value, median (IQR)	6.54 (5.40-9.51)	6.26 (5.27-9.48)	0.22 (0.59-0.21)
Value, range	2.79 to 14.51	2.25 to 14.07	–1.56 to 1.61
Value, 95% CI	5.48 to 9.92	5.22 to 9.99	–0.67 to 0.47

a Paired *t* test: *t*_11_=–0.370, *P*=.72 (not significant).

#### MFM Outcomes

##### Total MFM

Both the total scores and scores for each of the 3 dimensions in the MFM were analyzed. No significant differences were found in the total MFM score between the conventional and telerehabilitation approaches (*P*=.08). Telerehabilitation had an average decrease of 4% (95% CI −12% to 4%; Table 6).

**Table 6.** Results of the Motor Function Measure (MFM)^a^ (N=12).

Total MFM score	Conventional treatment	Telerehabilitation	Difference
Value, mean (SD)	0.89 (0.10)	0.85 (0.14)	–0.04 (0.13)
Value, median (IQR)	0.95 (0.84 to 0.97)	0.90 (0.77 to 0.96)	0.00 (–0.02 to 0.00)
Value, range	0.74 to 1.00	0.54 to 0.99	–0.44 to 0.02
Value, 95% CI	0.83 to 0.96	0.76 to 0.94	–0.12 to 0.04

a Paired Wilcoxon test: *V*=18.500; *P*=.08 (not significant).

##### D1: Standing and Transfer

No significant differences were found between the conventional and telerehabilitation approaches (*P*=.08). Telerehabilitation had an average decrease of 8% (95% CI −21% to 4%; Multimedia Appendix 3).

##### D2: Axial and Proximal Motor Function

No significant differences were found between the conventional and telerehabilitation approaches (*P*=.75). The scores remained stable at around 98% (95% CI −3% to 3%; Multimedia Appendix 4).

##### D3: Distal Motor Function

No significant differences were found between the conventional and telerehabilitation approaches (*P*=.89). Telerehabilitation had an average decrease of 4% (95% CI −15% to 7%; Multimedia Appendix 5).

### EPInfant Outcomes

Fatigue was measured for each session (before, in the middle of, and at the end of each session) during the telerehabilitation process. Both fatigue and fatigue evolution were estimated during each session. A clear evolution can be seen in the perception of fatigue throughout the 10 sessions.

#### EPInfant Within Sessions

**Figure 1.**
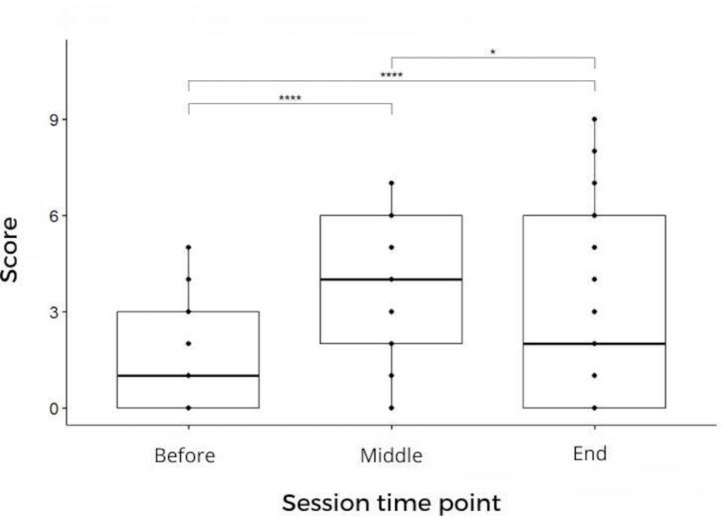
Effort Perception Infant (EPInfant) score variability within the sessions. Friedman test: *χ*^2^_2_=104.57, *P*<.001, n=120. Pairwise comparison by Wilcoxon test, with *P* values adjusted by Bonferroni correction. *P<.05 and ****P<.0001.

Infant effort significantly changed during the training sessions (*P*<.001; Table 7 and Figure 1). Fatigue peaked during the sessions but decreased by the end.

**Table 7.** Effort Perception Infant (EPInfant) score variability within the sessions.

Time point	Data points, n	EPInfant score			
		Range	Median (IQR)	Mean (SD; SE)	95% CI
Before the session	120	0-5	1 (0-3)	1.558 (1.549; 0.141)	1.278-1.838
In the middle of the session	120	0-7	4 (0-4)	3.633 (2.215; 0.202)	3.233-4.033
At the end of the session	120	0-9	2 (0-6)	3.117 (2.554; 0.233)	2.655-3.579

#### Evolution Throughout the Sessions

**Figure 2.**
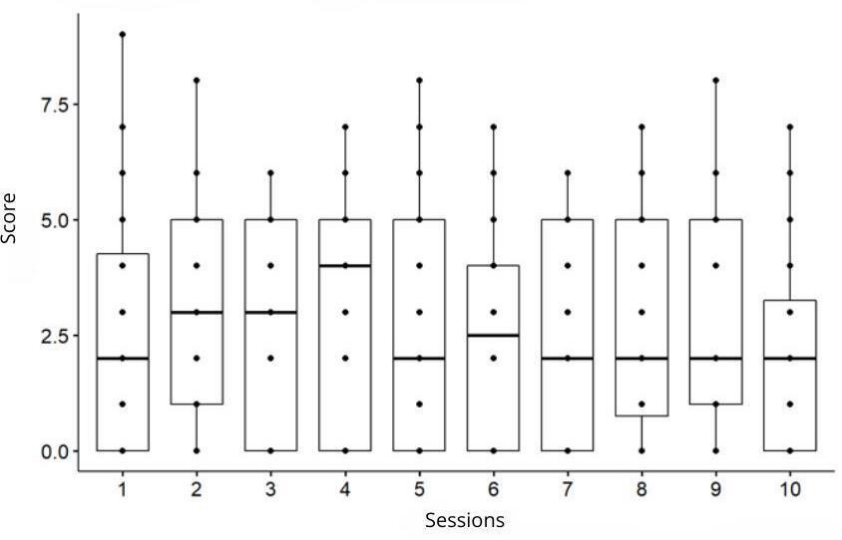
Fatigue perception evolution during the 10 sessions. Friedman test: *χ*^2^_9_=19.41, *P*=.02, n=36. Pairwise comparison by Wilcoxon test, with *P* values adjusted by Bonferroni correction.

Infant effort significantly changed throughout the training sessions (*P*=.02; Figure 2). The highest fatigue peaks occurred during the first session, and fatigue perception was lower starting in the third session, even though the exercise intensity actually increased.

The *P* value of the comparison between sessions 1 and 10 was statistically significant *(P*=.005), but the Bonferroni-adjusted *P* value was not (*P*=.24). Therefore, there was no significant comparison between the groups after the post hoc analysis.

#### Linear Mixed-Effects Model

**Figure 3.**
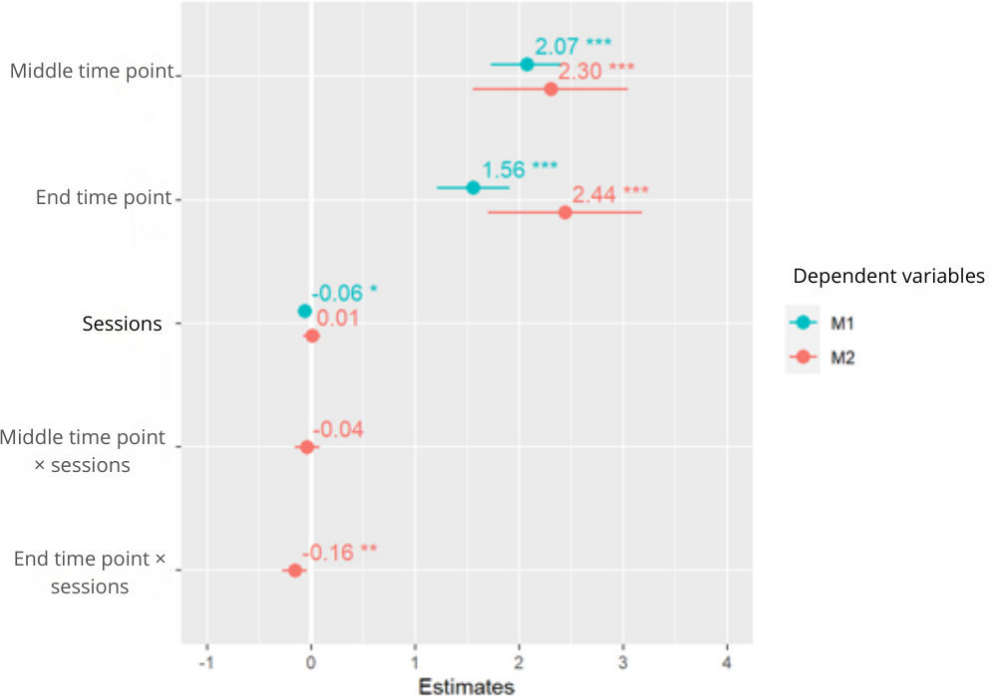
Linear mixed-effects models for the perception of fatigue. M1: model 1; M2: model 2. *P<.05 and ***P<.001.

There were significant differences between the different times and sessions (*P*<.05; Figures3  4). The perception of fatigue decreased as the number of sessions increased; the perception of fatigue at the middle and end time points within the sessions was higher than at the beginning.

**Figure 4.**
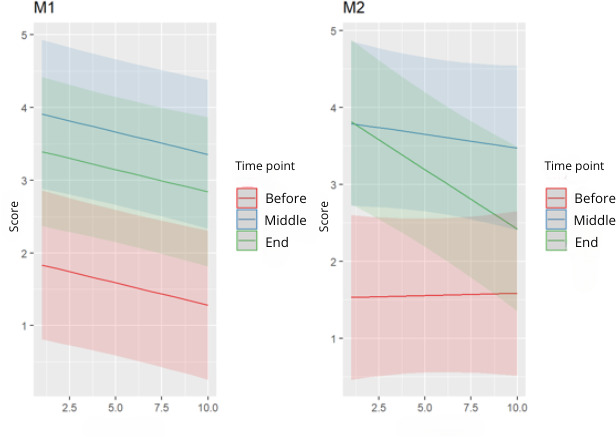
Comparison of 2-to-2 beats (comparing the start of the session to the middle, the middle of the session to the end, and the start of the session to the end. M1: model 1; M2: model 2.

### Fatigue Versus Motor Function Correlation

The Pearson correlation coefficient estimates the linear relationship between 2 continuous quantitative variables. The correlation is interpreted as follows: a positive correlation indicates a direct linear relationship between the 2 variables, whereas a negative correlation indicates an inverse linear relationship. The absolute value of the correlation indicates the strength of this association: 0-0.25=no correlation or very low correlation; 0.25-0.50=the association is weak; 0.50-0.75=the association is moderate to strong; and 0.75-1=the association is strong to perfect.

**Figure 5.**
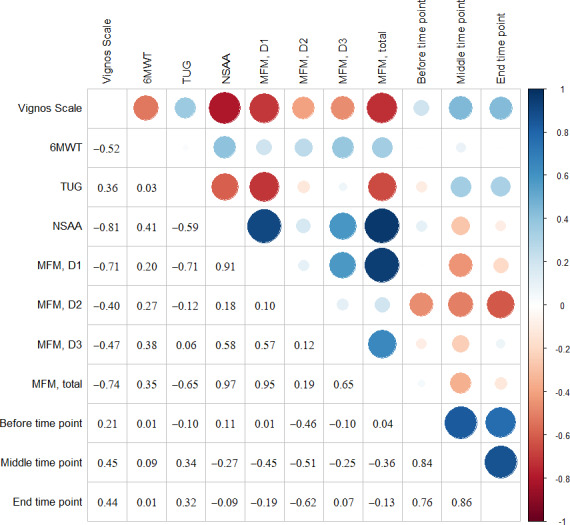
Correlation matrix between fatigue data (session 1) and motor function (conventional treatment). 6MWT: 6-minute walk test; MFM: Motor Function Measure; NSAA: North Star Ambulatory Assessment; TUG: timed up and go test.

In Figures5  6, the correlation values are shown in the lower-left triangle, and in the upper-right triangle, these values are depicted graphically. Blue color represents a positive relationship, whereas red color represents a negative relationship; furthermore, a larger circle represents a higher correlation.

**Figure 6.**
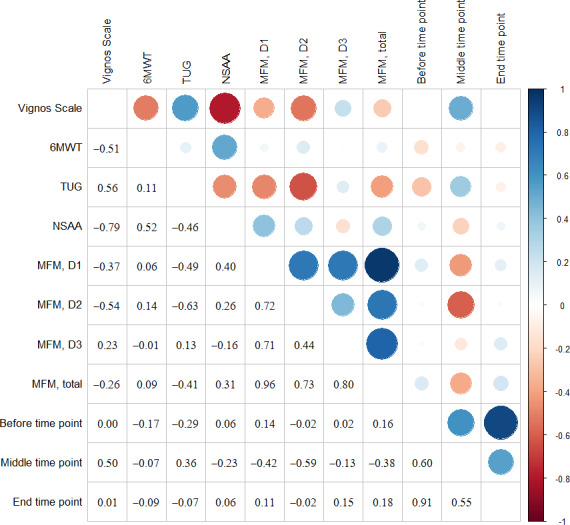
Correlation matrix between fatigue data (session 10) and motor function (telerehabilitation). 6MWT: 6-minute walk test; MFM: Motor Function Measure; NSAA: North Star Ambulatory Assessment; TUG: timed up and go test.

## Discussion

### Principal Findings

Considering telerehabilitation as a tool for physical therapy in children with DMD and BMD, the results of the functional tests did not show a significant change after 5 weeks of intervention. Although the 6MWT showed a 19.55-meter increase in the distance walked, this finding is not enough to confirm that one treatment is better than the other. However, it does help us understand that by conducting targeted training with children to carry out the test, its results are improved by decreasing the feeling of fatigue throughout the sessions.

The scores for the rest of the functional tests were the same before and after the program. Since the study was about progressive diseases, we can say that it is safe to carry out this type of program for short periods and that the motor skills of the children in the study did not get worse, as shown by the maintenance of the average score on the Vignos Scale.

### Comparison to Prior Work

The study of the efficacy of physiotherapy techniques within the framework of neuromuscular diseases is very broad. In a recent systematic review [28], an analysis was carried out on the quality of the studies applied in DMD and the field of study, obtaining some enlightening results on the disease. There are studies on the quality of life and studies that show data on the costs of the disease and mortality [28]. Furthermore, there are studies of the progression and prevalence of the disease and treatment guidelines [4,21,24].

The COVID-19 pandemic physically affected children with DMD and BMD. The study conducted by Tunç et al [29] on the parents of 272 children confirms the significant effects on both the medical care and the patients’ physical health. The reason the children did not attend physical therapy centers at the time was concern for contracting COVID-19. Most children did not follow physical therapy throughout the pandemic, and only 6% of the interviewed families had access to a telerehabilitation program at home.

It is well known that an established, continuous physical activity program is needed to maintain functioning in these types of dystrophy. Although the sample of our study was minimal and not limited to the Spanish territory, some of the children lived in towns far from resources and only received therapy once a month. In contrast, other children received weekly physical therapy and other therapies such as hydrotherapy.

Being able to bring physical therapy closer to these children is an achievement within the framework of telemedicine, which should be applied on a more regular basis. In fact, no patients abandoned the therapy during this study. In addition, we observed that the participants learned while performing the exercises, leading to decreased fatigue during the 10 sessions.

The main variable considered in this study was the 6MWT, since research for this disease considers it as a criterion for inclusion in pharmaceutical studies [30]. The NSAA also helped us predict the risk of the loss of ambulation of the participants [31].

Scientific literature supports the use of new technologies as a treatment for children with DMD. Thus, in 2019, de Freitas et al [32] compared performance in a virtual task using interfaces with and without physical contact to identify the use of different devices according to the functionality of the population with DMD. They found that leap motion provided better performance for people with DMD. Similarly, in 2020, Capelini et al [33] conducted a study with 50 individuals with DMD and a control group of 50 healthy individuals using a motor and visual task on a mobile phone. Performance improvement was obtained in a motor learning protocol using a mobile phone. The authors concluded that the use of technology could improve function in people with DMD.

In this study, since there was no control group, the 2 conditions were compared: when the children with DMD and BMD who could walk received the conventional treatment and when they received telerehabilitation. This differs from the study by Kenis-Coskun et al [34], which compared 2 groups with a sample of 19 children, where 1 group followed a telerehabilitation program and the other group performed exercises led through videos. Regarding the duration of the study, we conducted 2 sessions per week for 5 weeks, whereas Kenis-Coskun et al [34] conducted 8 sessions per week for 8 weeks. That study concludes that the telerehabilitation approach led to improvements compared to the group that performed exercises autonomously through the video program. Their conclusion reinforces our opinion that even in telerehabilitation, the presence of the physical therapist during the sessions to adjust the exercises helps to maintain functioning in children with DMD and BMD. The stretching-based program did not lead to clear results that could be implemented in future studies. However, we added aerobic exercises and the child’s own work at submaximal levels of physical effort, and the results showed that the functional capacities of these children did not decrease, even though the 19.55-meter increase in the 6MWT was not significant enough to conclude whether the program duration was long enough. In future studies, the programs should be extended for several months to show clearer results.

This program included respiratory exercises that are meant to be fun and designed for autonomous practice. However, some of them needed some family support, mainly to maintain the attention needed to do them. All the children who participated completed the exercises independently and found them to be fun. Sobierajska-Rek et al [5] conducted a study in 2021 where 45 children were instructed to do some exercises at home and then answer a survey. The caregivers explained that the exercises could be carried out at home, although most of the children needed help to be able to do them.

### Strengths and Limitations

A positive aspect that we found after carrying out the web-based program is its acceptance by children and their families. All the participants completed the 10 sessions at the indicated time.

The main limitation of this study was the small sample size. Since DMD and BMD are rare diseases, the eligible population was scarce. Furthermore, as the aim was to enroll children who could walk to perform the exercises while guided by the physical therapist as autonomously as possible, many children could not participate due to related cognitive and attention problems.

The second limitation was that we were unable to obtain significant differences between the variables measured. Future studies should assess maintaining the program for a longer time to see if there are significant differences.

The third limitation was regarding the economic aspect. Carrying out the study with glasses that could measure other parameters, such as heart rate, would have made it easier to measure fatigue during the exercises instead of relying on the child’s own perception. However, such glasses would be more expensive to send 1 to each participant, limiting participation to those who have a specific type of glasses.

### Future Directions

Telerehabilitation should be considered as an additional tool that has come to stay and should be implemented as routine therapy [35]. Since muscular dystrophy is a progressive disease, other types of interventions that manage the maintenance of motor functions instead of their improvement should be considered for future long-term studies. This type of program could be carried out in web-based groups, facilitating fun during physiotherapy training and not just passively stretching the muscles. It would initiate more conscious and active work at home by the child and their caregivers.

### Conclusions

Telerehabilitation is a useful tool to bring physical therapy to children who cannot go to a specific place to follow it. Although motor function as measured through the applied scales generally remained the same in the study, we can conclude that there were no significant differences between a conventional physical therapy treatment and a telerehabilitation program applied for 5 weeks. Therefore, this tool could be used without harming children with DMD and BMD, facilitating their access to therapies and stimulating learning to maintain their functional capacity. The learning effect helped reduce the feeling of fatigue in the children during the program.

Future studies should be carried out with a larger sample size and for a more extended period, to show whether a telerehabilitation program sustained over time helps maintain functioning in children with DMD and BMD and perhaps compare the results obtained between children with DMD and children with BMD.

## Supplementary material

10.2196/48022 Multimedia Appendix 1 Characteristics of the participants.

10.2196/48022 Multimedia Appendix 2 CONSORT (Consolidated Standards of Reporting Trials) flow diagram.

10.2196/48022 Multimedia Appendix 3 Results of the Motor Function Measure (MFM), D1: Standing and Transfer.

10.2196/48022 Multimedia Appendix 4 Results of the Motor Function Measure (MFM), D2: Axial and Proximal Motor Function.

10.2196/48022 Multimedia Appendix 5 Results of the Motor Function Measure (MFM), D3: Distal Motor Function.

## Abbreviations

6MWT: 6-minute walk test
BMD: Becker muscular dystrophy
CONSORT: Consolidated Standards of Reporting Trials
DMD: Duchenne muscular dystrophy
EPInfant: Effort Perception Infant
MFM: Motor Function Measure
NSAA: North Star Ambulatory Assessment
TUG: timed up and go test
VR: virtual reality
