# Supplementary figures and images for: Effectiveness of a 5-Week Virtual Reality Telerehabilitation Program for Children With Duchenne and Becker Muscular Dystrophy: Prospective Quasi-Experimental Study
Source: JMIR Serious Games. 2023 Nov 15;11:e48022. doi: 10.2196/48022 (PMC10686615; doi:10.2196/48022)

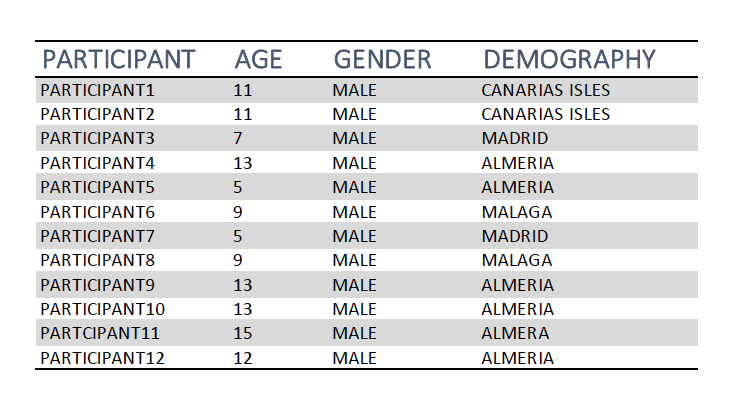

Supplement: Multimedia Appendix 1 [file games-v11-e48022-s001.png]
