# Supplementary material for: Effectiveness of a 5-Week Virtual Reality Telerehabilitation Program for Children With Duchenne and Becker Muscular Dystrophy: Prospective Quasi-Experimental Study
Source: JMIR Serious Games. 2023 Nov 15;11:e48022. doi: 10.2196/48022 (PMC10686615; doi:10.2196/48022)
Supplement: Multimedia Appendix 2 [file games-v11-e48022-s002.docx]

Enrollment

Assessed for eligibility (n=13)

Excluded (n= 1)

Not meeting inclusion criteria (n= 1)

Declined to participate (n=0)

Other reasons (n= 0)

Allocation

Randomized (n= 12)

Allocated to intervention time 1 (T1)

Received telerehabilitation program

Allocated to intervention time 2 (T2)

Did not receive allocated intervention

Evaluation at baseline (T1)

Evaluation after intervention (T2)

Follow-Up

Analysis

Statistical analysis of the different outcomes of study

Figure S1 CONSORT flow diagram of participants’ recruitment and progress through the phases of the trial. CONSORT, Consolidated Standards of Reporting Trials.
