# Supplementary material for: Effectiveness of a 5-Week Virtual Reality Telerehabilitation Program for Children With Duchenne and Becker Muscular Dystrophy: Prospective Quasi-Experimental Study
Source: JMIR Serious Games. 2023 Nov 15;11:e48022. doi: 10.2196/48022 (PMC10686615; doi:10.2196/48022)
Supplement: Multimedia Appendix 3 [file games-v11-e48022-s003.docx]

| **Variable** | **Conventional (N = 12)** | **Telerehabilitation (N = 12)** | **Difference (N = 12)** | **Test** | **Statistical** | ***P* value** | **Significance** |
| --- | --- | --- | --- | --- | --- | --- | --- |
| **MFM_32_D1** |  |  |  | Paired Wilcoxon | V = 14.000 | *P*=.08 | Non-significant |
| **- N** | 12 | 12 | 12 |  |  |  |  |
| **- Average (DS)** | 0.80 (0.20) | 0.72 (0.25) | -0.08 (0.20) |  |  |  |  |
| **- Median (Q1, Q2)** | 0.87 (0.62, 0.96) | 0.86 (0.53, 0.88) | 0.00 (-0.06, 0.00) |  |  |  |  |
| **- Range** | 0.44 - 1.00 | 0.26 - 0.97 | -0.69 - 0.03 |  |  |  |  |
| **- Average (CI95%)** | 0.80 (0.67, 0.93) | 0.72 (0.56, 0.87) | -0.08 (-0.21, 0.04) |  |  |  |  |
