# Supplementary material for: Effectiveness of a 5-Week Virtual Reality Telerehabilitation Program for Children With Duchenne and Becker Muscular Dystrophy: Prospective Quasi-Experimental Study
Source: JMIR Serious Games. 2023 Nov 15;11:e48022. doi: 10.2196/48022 (PMC10686615; doi:10.2196/48022)
Supplement: Multimedia Appendix 4 [file games-v11-e48022-s004.docx]

| **Variable** | **Conventional (N = 12)** | **Telerehabilitation (N = 12)** | **Difference (N = 12)** | **Test** | **Statistical** | ***P* value** | **Significance** |
| --- | --- | --- | --- | --- | --- | --- | --- |
| **MFM_32_D2** |  |  |  | Paired Wilcoxon | V = 12.000 | *P*= .752 | Non-significant |
| **- N** | 12 | 12 | 12 |  |  |  |  |
| **- Average (DS)** | 0.98 (0.03) | 0.98 (0.04) | -0.00 (0.05) |  |  |  |  |
| **- Median (Q1, Q2)** | 1.00 (0.97, 1.00) | 1.00 (0.98, 1.00) | 0.00 (-0.01, 0.01) |  |  |  |  |
| **- Range** | 0.89 - 1.00 | 0.89 - 1.00 | -0.08 - 0.11 |  |  |  |  |
| **- Average (CI95%)** | 0.98 (0.96, 1.00) | 0.98 (0.95, 1.00) | -0.00 (-0.03, 0.03) |  |  |  |  |
