# Supplementary material for: Effectiveness of a 5-Week Virtual Reality Telerehabilitation Program for Children With Duchenne and Becker Muscular Dystrophy: Prospective Quasi-Experimental Study
Source: JMIR Serious Games. 2023 Nov 15;11:e48022. doi: 10.2196/48022 (PMC10686615; doi:10.2196/48022)
Supplement: Multimedia Appendix 5 [file games-v11-e48022-s005.docx]

| **Variable** | **Conventional (N = 12)** | **Telerehabilitation (N = 12)** | **Difference (N = 12)** | **Test** | **Statistical** | ***P* value** | **Significance** |
| --- | --- | --- | --- | --- | --- | --- | --- |
| **MFM_32_D3** |  |  |  | Paired Wilcoxon | V = 8.000 | *P*=.893 | NS |
| **- N** | 12 | 12 | 12 |  |  |  |  |
| **- Average (DS)** | 0.96 (0.08) | 0.92 (0.16) | -0.04 (0.17) |  |  |  |  |
| **- Median (Q1, Q2)** | 1.00 (0.95, 1.00) | 0.98 (0.94, 1.00) | 0.00 (0.00, 0.01) |  |  |  |  |
| **- Range** | 0.71 - 1.00 | 0.43 - 1.00 | -0.57 - 0.10 |  |  |  |  |
| **- Average (CI95%)** | 0.96 (0.90, 1.01) | 0.92 (0.81, 1.02) | -0.04 (-0.15, 0.07) |  |  |  |  |
